# Supplementary material for: Pharmacist-Managed Therapeutic Drug Monitoring Programs within Australian Hospital and Health Services—A National Survey of Current Practice
Source: Pharmacy (Basel). 2022 Oct 18;10(5):135. doi: 10.3390/pharmacy10050135 (PMC9607166; doi:10.3390/pharmacy10050135)
Supplement: Supplementary file 1 [file pharmacy-10-00135-s001.zip › pharmacy-1938128-supplementary.pdf]

pharmacy

MDPI

Introduction

As part of a Research Higher Degree through the University of Queensland's School of Pharmacy, we are reviewing Therapeutic Drug Monitoring (TDM) programs and the role of pharmacists within these programs. How TDM is undertaken varies within hospitals and health services across Australia and the world. Although the process of when and for which drugs TDM should be performed is well discussed in the literature, parts of the process such as who should perform TDM and who should interpret the results is varied and is not well discussed.

Purpose

The purpose of this survey is to obtain some information about current TDM programs and the role of the pharmacist within those programs within hospitals and health services across Australia.

Consent

By clicking the link to the survey and completing the survey I provide my consent to participate.

- I understand that I am being asked to participate in a survey that forms part of Doctor of Philosophy thesis through the University of Queensland. It is my understanding that this survey has been designed to gather information about Therapeutic Drug Monitoring Programs within Hospitals and Health Services and the pharmacist's role within them.
- I have read the Participant Information Sheet for this study and understand the reasons for the survey and the types of information being collected.
- I understand that my participation in this project is completely voluntary and that I am free to decline to participate, without consequence, at any time prior to, or at any point during, the survey.
- I understand that any information I provide is anonymous, will be kept confidential, and used only for the purposes outlined within the Participant Information Sheet. All survey responses will be kept in a secured environment.
- I also understand that there are no risks involved in participating in this activity, beyond those risks experienced in everyday life.

<u>Section One: Questions - Introduction</u>

<u>Please answer the following questions by selecting the answer that applies to you</u>

1. What State or Territory are you located in?
    a. Queensland
    b. New South Wales
    c. Australian Capital Territory
    d. Victoria
    e. Tasmania
    f. South Australia
    g. Northern Territory
    h. Western Australia

2. What type of hospital do you currently work in?
    a. Public Hospital Metropolitan
    b. Public Hospital Rural
    c. Private Hospital Metropolitan
    d. Private Hospital Rural
    e. Other, please specify

3. How many acute inpatient beds are available within your hospital?
    a. <100
    b. 101-500
    c. 501-1000
    d. >1001

4. Are you a digital hospital? (I.e. digital medication charts, digital progress notes, digital ordering systems for pathology, radiology etc.)
    a. Yes
    b. No

5. Does your hospital have a clinical pharmacology service (medical officers with specialist training in clinical pharmacology)?
    a. Yes – within the hospital/site
    b. Yes –but not located within the hospital/site
    c. No

6. Does your hospital have onsite pathology with the ability to process Therapeutic Drug Monitoring?
    a. Yes – a full pathology service with all Therapeutic Drug Monitoring
    b. Yes – only limited Therapeutic Drug Monitoring available. All other Therapeutic Drug Monitoring are processed off-site
    c. No

7. Does your hospital have a pharmacist managed Therapeutic Drug Monitoring program?
    a. Yes – Section Three
    b. No – Section Two

Section Two

8. Who is responsible for ordering Therapeutic Drug Monitoring when it is required?
    a. Doctors
    b. Nurses
    c. Pharmacists
    d. Pathology Staff
    e. Other, please specify …………………………………………………………..

9. Who is responsible for actioning Therapeutic Drug Monitoring results?

a. Doctors

b. Nurses

c. Pharmacists

d. Pathology Staff

e. Other, please specify ………………………………………………………………………….

10. Do you currently experience any of the following problems around Therapeutic Drug Monitoring at your hospital? (Please answer each item)

a. Inappropriately timed sample collection

Never	Rarely	Sometimes	Often	Always
1	2	3	4	5

b. No samples being collected

Never	Rarely	Sometimes	Often	Always
1	2	3	4	5

c. Inappropriate actioning of results

Never	Rarely	Sometimes	Often	Always
1	2	3	4	5

d. No actioning of results from therapeutic drug monitoring assays

Never	Rarely	Sometimes	Often	Always
1	2	3	4	5

e. We don’t experience any problems with Therapeutic Drug Monitoring

Never	Rarely	Sometimes	Often	Always
1	2	3	4	5

f. Other, please specify

11. Do you believe there are benefits to a pharmacist managed Therapeutic Drug Monitoring program?
    a. Yes (go to question 12)
    b. No (go to question 13)

12. If yes, what do you believe the benefits are? (please select all that apply)
    a. Better patient outcomes
    b. Expanded scope of the pharmacist
    c. More engagement with the health facility (i.e. management and executive)
    d. More engagement with multidisciplinary team
    e. Other, please specify

13. If no, why do you not think there are benefits? (please select all that apply)
    a. Impacts on current workload
    b. Disruption to workflows
    c. Out of scope of pharmacists
    d. Costs
    e. Other, please specify

14. What do you believe are the barriers within your health facility in having a pharmacist managed Therapeutic Drug Monitoring program? (please select all that apply)
    a. Lack of external staff engagement
    b. Lack of internal staff engagement
    c. Lack of funding for the service delivery
    d. Time
    e. Other (Please specify)

15. What medications would you like to see included in a pharmacist managed Therapeutic Drug Monitoring program? (please select all that apply)
    a. Glycopeptide Antibiotics (i.e. Vancomycin)
    b. Aminoglycosides (i.e. Gentamicin)
    c. Low Molecular Weight Heparin
    d. Unfractionated Heparin
    e. Warfarin
    f. Direct Oral Anticoagulants (i.e. rivaroxaban, apixaban)
    g. Clozapine
    h. Digoxin
    i. Anti-epileptics (i.e. phenytoin, carbamazepine, sodium valproate)
    j. Other – please specify

Section Three

16. What medications are including in your pharmacist managed Therapeutic Drug Monitoring program? Please select all that apply
    a. Glycopeptide Antibiotics (i.e. Vancomycin)
    b. Aminoglycosides (i.e. Gentamicin)
    c. Low Molecular Weight Heparin
    d. Unfractionated Heparin
    e. Warfarin
    f. Direct Oral Anticoagulants (i.e. rivaroxaban, apixaban)
    g. Clozapine
    h. Digoxin
    i. Anti-epileptics (i.e. phenytoin, carbamazepine, sodium valproate)
    j. Others – please specify

17. What part of therapeutic drug monitoring is the pharmacist responsible for? (Please select more than one if required)

a. Recommending to medical staff when Therapeutic Drug Monitoring should occur

Never	Rarely	Sometimes	Often	Always
1	2	3	4	5

b. Alerting medical staff when Therapeutic Drug Monitoring results are available

Never	Rarely	Sometimes	Often	Always
1	2	3	4	5

c. Ordering blood tests for Therapeutic Drug Monitoring if required

Never	Rarely	Sometimes	Often	Always
1	2	3	4	5

d. Calculating subsequent doses

Never	Rarely	Sometimes	Often	Always
1	2	3	4	5

e. Prescribing subsequent doses

Never	Rarely	Sometimes	Often	Always
1	2	3	4	5

f. Other (please specify)

18. Do your pharmacists have a credentialing or education package they must complete prior to undertaking Therapeutic Drug Monitoring?

a. No

b. Yes – an in-house program

c. Yes – conducted externally from the pharmacy

d. Other, please specify ………………………………………………………………………………..

19. How often are the pharmacists required to undertake the credentialing or education package?
    a. Never
    b. Once
    c. Yearly
    d. Every 2 years
    e. Other (please specify)

20. How is the credentialing or education package delivered?
    a. Self-directed learning
    b. By the department clinical educator
    c. By a senior/specialist pharmacist
    d. External person
    e. External Body (Please specify)
    f. Another Internal Person (Please specify)
    g. Other, please specify …………………………………………………………

21. Are pharmacists required to be a certain number of years' post registration to be involved in the pharmacist managed therapeutic drug monitoring program?
    a. Yes
    b. No (go to question 23)

22. If yes, how many years?
    a. 1
    b. 2
    c. 3

d. >5 years

23. Does the pharmacist require a post-graduate qualification to take part in the pharmacist managed Therapeutic Drug Monitoring program?
    a. Yes (please specify) ……………………………………………….
    b. No

24. What are the benefits of a pharmacist managed Therapeutic Drug Monitoring program?
    a. Better patient outcomes
    b. Expanded scope of the pharmacist
    c. More engagement with the health facility (i.e. management and executive)
    d. More engagement with multidisciplinary team
    e. Other (please specify)

25. What are the negative aspects of the pharmacist managed Therapeutic Drug Monitoring program?
    a. Added workload for pharmacists
    b. Difficulty maintaining credentialing and education packages
    c. Lack of experienced staff to participate in the program
    d. Cost
    e. Other (please specify)

26. Are there any other comments you would like to make about pharmacist managed Therapeutic Drug Monitoring? Please use the following space for your response.

Thank you for your time
